# The TRPM4 channel inhibitor 9-phenanthrol alleviates cerebral edema after traumatic brain injury in rats

**DOI:** 10.3389/fphar.2023.1098228

**Published:** 2023-02-14

**Authors:** Ping Ma, Ning Huang, Jun Tang, Zunjie Zhou, Jing Xu, Yi Chen, Maoxin Zhang, Qin Huang, Yuan Cheng

**Affiliations:** ^1^ Department of Neurosurgery, The Second Affiliated Hospital of Chongqing Medical University, Chongqing, China; ^2^ Department of Rehabilitation, The Second Affiliated Hospital of Chongqing Medical University, Chongqing, China

**Keywords:** traumatic brain injury, cerebral edema, 9-phenanthrol, TRPM4, MMP-9, PI3K/AKT/NF-kB signaling pathway

## Abstract

Cerebral edema (CE) exerts an important effect on brain injury after traumatic brain injury (TBI). Upregulation of transient receptor potential melastatin 4 (TRPM4) in vascular endothelial cells (ECs) results in damage to capillaries and the blood-brain barrier (BBB), which is critical for the development of CE. Many studies have shown that 9-phenanthrol (9-PH) effectively inhibits TRPM4. The current study aimed to investigate the effect of 9-PH on reducing CE after TBI. In this experiment, we observed that 9-PH markedly reduced brain water content, BBB disruption, proliferation of microglia and astrocytes, neutrophil infiltration, neuronal apoptosis and neurobehavioral deficits. At the molecular level, 9-PH significantly inhibited the protein expression of TRPM4 and MMP-9, alleviated the expression of apoptosis-related molecules and inflammatory cytokines, such as Bax, TNF-α and IL-6, near injured tissue, and diminished serum SUR1 and TRPM4 levels. Mechanistically, treatment with 9-PH inhibited activation of the PI3K/AKT/NF-kB signaling pathway, which was reported to be involved in the expression of MMP-9. Taken together, the results of this study indicate that 9-PH effectively reduces CE and alleviates secondary brain injury partly through the following possible mechanisms: ①9-PH inhibits TRPM4-mediated Na + influx and reduces cytotoxic CE; ②9-PH hinders the expression and activity of MMP-9 by inhibiting the TRPM4 channel and decreases disruption of the BBB, thereby preventing vasogenic cerebral edema. ③9-PH reduces further inflammatory and apoptotic damage to tissues.

## Introduction

Cerebral edema (CE), characterized by excessive water accumulation in brain cells and extracellular spaces, is one of the most important secondary pathologies that occurs after traumatic brain injury (TBI) *(* Xiong et al., 2013; Jha et al., 2019 *).* To date, the mechanism underlying CE after TBI is very complex and not fully understood. Typically, inflammatory reactions, oxidative stress, mitochondrial dysfunction and changes in different ion channels participate in the progression of CE (Faden, 2002; Jassam et al., 2017; Jha et al., 2019 *).* Increased brain volume due to oncotic tissue eventually leads to increased intracranial pressure (ICP) and is even followed by the development of cerebral hernia, which almost always causes death in patients in the clinic (Huttner and Schwab, 2009). Most importantly, only passive surgical decompression and dehydration therapy, which have poor efficacy, can be used to treat edema, and an efficient and specific strategy is urgently needed (Adukauskiene et al., 2007; Hackenberg and Unterberg, 2016).

Transient receptor potential melastatin 4 (TRPM4) is a voltage dependent, non-selective cation channel that is mainly permeable to Na+, activated by elevated cytosolic Ca2+, and modulated by ATP; TRPM4 is exceptional in that it is one of only two known ion channels in the mammalian genome that conduct monovalent cations exclusively and non-selectively (Vennekens and Nilius, 2007; Guinamard et al., 2011). TRPM4 is expressed at low levels and is mainly involved in the regulation of vasoconstriction and respiratory rhythm; its expression is restricted to arteries and the brainstem in the central nervous system (CNS). Under pathological conditions, such as stroke, it is upregulated and combines with sulfonylurea receptor 1 (SUR1) to form the SUR1-TRPM4 channel complex (Guinamard et al., 2011). Previous studies have shown that SUR1-TRPM4 is overexpressed in all neurovascular unit members (including neurons, endothelial cells (ECs), astrocytes and microglia) and first appears in ECs in the early stage of CNS injury (Makar et al., 2015; King et al., 2018; Chen et al., 2019b; Li et al., 2020). It is well known that disruption of the blood‒brain barrier (BBB) dominates the development of vasogenic CE (Chen et al., 2021). The TRPM4-mediated influx of large amounts of sodium ions stimulates endothelial cell swelling and death and thereby triggering the disruption of the BBB. In addition, although the specific mechanism is unknown, SUR1-TRPM4 has been reported in several studies to be closely related to the expression of matrix metalloproteinase-9 (MMP-9), which is able to degrade the BBB (Jiang et al., 2017; Gerzanich et al., 2018). Interventions that negatively regulate SUR1-TRPM4, including the SUR1 inhibitor glibenclamide (GLC), siRNA or polyclonal antibody targeting TRPM4, have exhibited favorable curative effects in decreasing secondary CE and promoting BBB integrity and neural function recovery (Jiang et al., 2017; Gerzanich et al., 2018; Chen et al., 2019a; Chen et al., 2019b; Chen et al., 2022). These results suggest that SUR1-TRPM4 inhibition has great potential in improving the prognosis of stroke, TBI and other CNS diseases. At present, GLC is widely studied as an inhibitor of SUR1-TRPM4. Although many achievements have been made, the latest clinical research results demonstrated that GLC at a safe dose could not significantly improve the prognosis of stroke patients (Sheth et al., 2016), exemplifying the limitations of GLC treatment and showing the importance of developing new treatments that target TRPM4.

As a recently discovered TRPM4 inhibitor, 9-phenanthrol (9-PH) has greater specificity for TRPM4 than non-steroidal anti-inflammatory drugs, such as flufenamic acid (FFA), spermine and quinine (Guinamard et al., 2014). *In vitro* studies have shown that 9-PH can block 80%–90% of TRPM4 currents in many cells (such as HEK-293 cells, rat cerebral vascular smooth muscle cells, monkey colon smooth muscle cells and mouse smooth muscle cells) (Grand et al., 2008; Amarouch et al., 2013; Guinamard et al., 2014). Encouragingly, 9-PH was also shown to inhibit TRPM4 in neurons cultured *in vitro* (Mrejeru et al., 2011; Guinamard et al., 2014). However, whether 9-PH can reduce CE and tissue damage after CNS injury by inhibiting TRPM4 *in vivo* is still unknown. Recently, a study reported that 9-PH could restore cerebral blood flow by inhibiting TRPM4 after subarachnoid hemorrhage in rats (Gong et al., 2019). Therefore, based on previous research, we hypothesized that 9-PH might be a potential therapeutic agent in TBI.

In this study, we first applied 9-PH to treat TBI and aimed to investigate the effect and mechanism of 9-PH on reducing CE and promoting neural function after TBI. Then, we examined the neurobehavioral deficits and brain water content, BBB permeability, TRPM4 expression and MMP-9 activation, and the inflammatory immune response triggered by tissue damage and BBB disruption. The results indicated that 9-PH treatment significantly decreased the expression of TRPM4 and MMP-9 and alleviated the MMP-9-mediated damage to the BBB by inhibiting the activation of the PI3K/AKT/NF-kB signaling pathway, thereby reducing tissue edema and improving neurological deficits after TBI in rats.

## Materials and methods

### Animal preparation and groups

Adult male Sprague‒Dawley rats weighing 250–300 g were purchased and raised at the Animal Center of Chongqing Medical University. All the rats were housed in a controlled environment (12/12 h light/dark cycle, 22°C–25°C, 55%–65% humidity) for at least 5 days and allowed to adapt to the environment; the rats had free access to food and water. The animals were randomly divided into five groups: the sham-operated group (sham group), TBI + non-intervention group (TBI group), TBI + vehicle (DMSO) (vehicle group), TBI + 9-PH (70 μg/kg) (9-PH group), and TBI + PI3K inhibitor (LY294002 group). A total of 168 rats were included in this study, and nine rats were excluded due to death or abnormal operation during the experiment. All the procedures involving animals conformed to the Guide for the Care and Use of Laboratory Animals of the National Institutes of Health and were approved by the Institutional Animal Care and Use Committee of Chongqing Medical University.

### Traumatic brain injury rat model induction

The traumatic brain injury (TBI) model was established using the modified Allen method as previously described (Feeney et al., 1981). In brief, rats were anesthetized by 2% pentobarbital sodium (2 mL/kg, i.p.) and fixed in a prone position in a stereotaxic frame. Lidocaine plus adrenaline (2000:1) was injected under the scalp to reduce bleeding. A 1 cm midline incision was made on the skull to separate the skin and fascia. Then, a craniotomy with a diameter of 4 mm was drilled at 2 mm to the right and post of the bregma, ensuring the integrity of the dura mater. A 10 g weight was dropped along the vertical pipe at 20 cm height, hitting the impactor to cause a 1 mm deep traumatic brain injury. The weight remained on the impactor for 60 s, and then, the impactor was removed. The sham-operated rats underwent the same procedures, but the impact on the cortex was omitted.

### 9-Phenanthrol administration

9-Phenanthrol (9-PH) was obtained from Yuanye Bio-Technology (S7263, Shanghai, China), dissolved in dimethyl sulfoxide (DMSO) to obtain a 20 mM stock solution, stored in separate aliquots at −80°C, and then diluted with unbuffered saline (0.9% NaCl) to 60 μM for research. The concentration of DMSO was less than 0.5%. 9-PH (70 μg/kg) was intraperitoneally injected 15 min after the operation once per day for seven consecutive days. In the TBI + Vehicle group and sham-operated group, the same solution without 9-PH was injected in the same way. For the TBI + PI3K inhibitor group, LY294002 (1 mg/kg per day) was injected i.p. 15 min after injury. The TBI + non-intervention group received no injections.

### Brain water content

As previously described, cerebral edema was determined by measuring the brain water content 24 h after TBI *via* a researcher who was blinded to group information (Fu et al., 2020). Briefly, rats were decapitated under deep anesthesia. The brain was immediately removed, and the injured hemisphere was rapidly weighed on an electronic analytical balance (FA2204B, Techcomp, United States) to determine the wet weight (WW) and then dried at 60°C for 7 days to determine the dry weight (DW). Brain water content (%) was calculated using the following formula: brain water content (%) = [(wet weight −dry weight)/wet weight] × 100%.

### Neurobehavioral function test

Neurobehavioral functions were evaluated using the modified Garcia test and corner turn test at 24 h following TBI by a blinded investigator as previously described (Altay et al., 2012; Xie et al., 2017). In the modified Garcia test, six items, including spontaneous activity, spontaneous movement of all four limbs, forepaw outstretching, body proprioception, response to vibrissae touch, and climbing, were measured. Each item was scored as either 0–3 or one to three, and the total scores ranged from 3 to 18. A higher score indicated better neurological function. In the corner turn test, the rats were allowed to approach a 30° corner. The rats exited the corner with either a right turn or a left turn. Fifteen trials were performed, with at least a 30-s break between the trials. The percentage of right turns in 15 trials was then calculated.

### Enzyme-linked immunosorbent assay

For the quantitative determination of serum protein levels, rats were sacrificed at the scheduled experimental time points (0 h, 6 h, 12 h, 24 h, 48 h, 72 h, 120 h and 168 h after injury). Then, the thoracic cavities were dissected and exposed, and 2 mL blood was collected from the heart into a tube without anticoagulant. The blood was preserved at 4°C for 1 h and then centrifuged (4°C, 4,000 rpm, 10 min), and the serum was frozen at −80°C. Commercial rat SUR1 ELISA kits (QZ-22516, JiuBang Biotech, Fujian, China) and rat TRPM4 ELISA kits (QX- 24726, JiuBang Biotech, Fujian, China) were used for the test, and the procedure was carried out according to the instructions. The protein concentration was measured by a spectrophotometer (Thermo Fisher Scientific, United States). Three multiple wells were established for each sample, and the standard curve was drawn according to the OD value of the standard samples. The average concentration of the three multiple wells represented the actual concentration of the sample.

### Blood‒Brain barrier permeability

The permeability of the blood‒brain barrier (BBB) was investigated by Evans blue (E808783, Macklin, China) extravasation as previously described with some modifications (Xu et al., 2015). After anesthetizing the rats, 3% Evans Blue solution (3 mL/kg) was injected into the rats through the femoral vein and allowed to circulate for 1 h. Then, the rats were transcardially perfused with cold phosphate-buffered saline (0.1 M, PBS, pH 7.4) under deep anesthesia until the outflow became clear and transparent. The brain was collected and immediately stored at −80°C. Each sample of ipsilateral brain was weighed and homogenized in PBS (500 μL PBS for every 100 mg tissue), sonicated, and centrifuged (12,000 g, 4°C, 30 min). The supernatant was collected, mixed with an equal volume of trichloroacetic acid (TCA), and then incubated overnight at 4°C. After centrifugation (12,000 g, 4°C, 30 min), Evans blue staining was determined at 610 nm with a spectrophotometer (Thermo Fisher Scientific, United States).

### Tissue preparation

Rats were executed under deeply anesthetized at 24 h after the injury, then precooled PBS (0.1 M, pH 7.4) and 4% paraformaldehyde was successively perfused *via* ventricle. Then the brain was dissected and separated. Tissue blocks with a diameter of 0.5 cm containing the injury center were cut, and immersed in 4% paraformaldehyde fixative for 48 h. Then rinsed in PBS overnight. For frozen sections, rinsed tissue was placed in 30% sucrose (dissolved in PBS), dehydrated overnight at 4°C, and then buried in OCT. Blocks were sliced along the coronal plane on the cryostat (CM 1860; Leica, Germany) with a thickness of 10 um. The frozen sections were immediately stored at −20°C. For paraffin sections, after rinsing, the tissues were dehydrated with ethanol (50%, 75%, 85%, 90%, 95%, absolute) for 20 min per gradient, and then embedded with paraffin, sliced with a section thickness of 5 um. For molecular research, the ipsilateral tissues were dissected and separated after perfusion of precooled PBS, and then preserved at −80°C.

### Gelatin zymography

Fresh brain tissues were immersed in RIPA lysis buffer (P0013K, Beyotime, China) at a ratio of 1:4 (w: v) and centrifuged at 4°C (12,000 rpm, 15 min) after homogenization. The protein concentration was determined by a BCA concentration determination kit (P0010, Beyotime, China). The supernatant and loading buffer (P0016N, Beyotime, China) were mixed at a ratio of 4:1. A total of 120 μg of total protein was loaded into an 8% SDS‒PAGE gel containing 10% gelatin. After electrophoresis at 80 V in ice water for 3 h, each gel was washed in 2.5% Triton X-100 for 30 min 3 times. Then, gels were treated with incubation buffer (50 mM Tris-HCL, 0.2 M NaCl, 5 mM CaCl2, 1 µM ZnCl2, 0.02% Brij-35, pH 7.5) for 48 h at 37°C on a shaking table. Gels were stained with 0.5% Coomassie brilliant blue R250 (ST1123, Beyotime, China) for 3 h and then dyed with 30% methanol containing 10% acetic acid until the emergence of an appropriate color discrepancy. The clear bands on the zymogram represented gelatinase activity.

### Western blotting

Western blotting was performed as previously described (Luo et al., 2020). Separated tissue was immersed in RIPA lysis buffer (P0013B, Beyotime, China) at 1:7.5 (w: v) to prepare protein extract samples. The protein concentration was determined with a BCA concentration determination kit. An equal amount sample per lane was separated by SDS‒PAGE and transferred to PVDF membranes. The membranes were incubated with primary antibodies overnight at 4°C. The primary antibodies were anti-TRPM4 (1:500, PA5-77324, ThermoFisher, USA), anti-MMP-9 (1:1,000, ab283594, Abcam, United States), anti-ZO-1 (1:1,000, 21773-1-AP, Proteintech, China), anti-Occludin (1:1,000, 13409-1-AP, Proteintech, China), anti-Claudin-5 (1:1,000, A10207, Abclonal, China), anti-Claudin-1 (1:1,000, 13050-1-AP, Proteintech, China), anti-Bax (1:1,000, 50599-2-Ig, Proteintech, China), anti-Bcl2 (1:1,000, 26593-1-AP, Proteintech, China), anti-GFAP (1:1,000, #80788, CST, United States), anti-TNF-α (1:1,000, 346,654, Zen-bio, China), anti-IL-6 (1:1,000, A11115, Abclonal, China), anti-p-AKT (1:1,000, #4060, CST, USA), anti-AKT (1:1,000, #4685, CST, USA), anti-p-PI3K (1:1,000, #4228, CST, United States), anti-PI3K (1:1,000, #4292, CST, United States), anti-p-P65 (1:1,000, #3033, CST, USA), anti-P65 (1:1,000, #8242, CST, United States), anti-β-actin (1:5,000, 66009-1-Ig, Proteintech, China). Then, the membranes were incubated with a goat anti-rabbit or goat anti-mouse IgG secondary antibody at room temperature for 1 h. The ECL Plus chemiluminescence kit (Amersham Biosciences, Arlington Heights, PA, USA) was used to detect the bands, which were visualized by the image system (VersaDoc, 4,000 type, Bio Rad, Hercules, CA, USA). The relative density of proteins was analyzed with ImageJ software (ImageJ, RRID: SCR _ 003070).

### Immunofluorescence staining

Immunofluorescence staining was performed as previously described (Chen et al., 2020). In short, the frozen sections were rewarmed at room temperature. After antigen retrieval in citric acid antigen repair solution, the sections were incubated at 4°C overnight with the following primary antibodies: anti-CD31 (1:200, ab222783, Abcam, USA), anti-IBA-1 (1:400, 019-19741, FUJIFILM Wako Shibayagi, Japan), anti-CD68 (1:200, 28058-1-AP, Proteintech, China), anti-GFAP (1:400, #80788, CST, USA), anti-MPO (1:50, A1374, Abclonal, China), and anti-NeuN (1:200, #94403, CST, USA). Then, the sections were incubated with the appropriate fluorescently labeled secondary antibody at room temperature for 1 h. For double staining with TUNEL fluorescence, the procedure followed the instructions of the CF488 TUNEL Cell Apoptosis Detection Kit (G1504, Servicebio, Wuhan, China). After incubation with secondary antibody, the sections were equilibrated with Equilibrium Buffer for 10 min and then incubated at 37°C for 1 h with TDT incubation buffer. Finally, nuclei were stained with DAPI. The sections were observed and photographed with a fluorescence microscope (U-HGLGPS, Olympus, Japan). Micrographs were analyzed with ImageJ software (ImageJ, RRID: SCR _ 003070).

### Morphological quantification

Hemorrhage in brain samples was measured by hematoxylin and eosin (H&E) staining. Three sections of each brain were used for H&E staining. Hematoxylin staining was performed for 5 min, and eosin staining was performed for 30 s. After image acquisition, ImageJ software (ImageJ, RRID: SCR _ 003,070) was used to analyze the amount and range of bleeding. The average value of three slices represents the bleeding condition of each tissue.

NISSL staining was used to assess the number of surviving neurons. After dewaxing, the sections were stained with toluidine blue dye at 60°C for 1 h and then washed in double distilled water for 5 min 3 times. The average number of surviving neurons in the sections was calculated to reflect neuronal damage.

### Statistical analysis

All the data are expressed as the mean and standard deviation (mean +SD). GraphPad Prism eight software (GraphPad Prism, RRID: SCR _ 002,798) was used for all the analyses. The comparison between two groups (*n* ≤ 2) was performed by unpaired Student’s t test. For multiple comparisons (*n* ≥ 3), data were subjected to one-way ANOVA to determine the level of statistical significance. A *p*-value less than 0.05 was defined as statistically significant.

## Results

### 9-Phenanthrol treatment alleviated brain water content and neurological deficits

**FIGURE 1:**
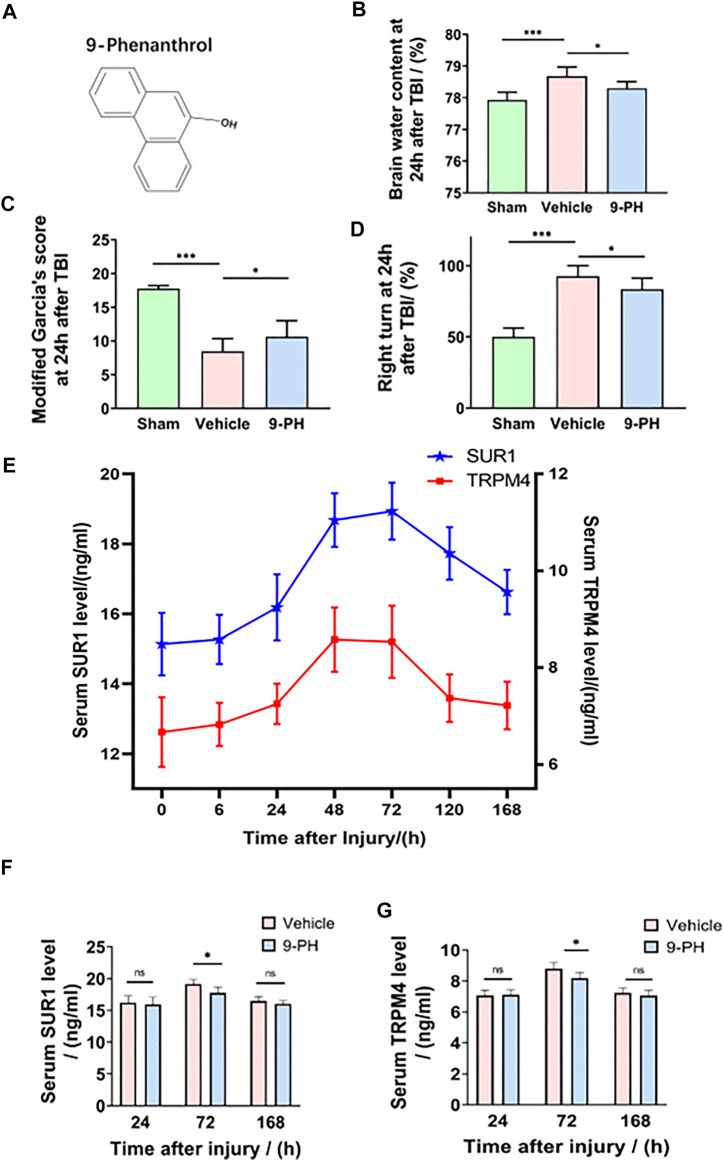
Neuroprotective effects of 9-PH on brain injury after TBI. **(A)** The molecular structure of 9-phenanthrol. **(B–D)** Brain water content of rat ipsilateral cerebral hemispheres and neurological deficit analysis of rats 24 h after injury (*n* = 8). **(E)** Line chart of the trend of change in serum SUR1 and serum TRPM4 levels at 0 h, 6 h, 24 h, 48 h, 72 h, 120 h and 168 h after TBI (*n* = 6 for each point). **(F, G)** Analysis of SUR1 and TRPM4 levels in the serum of rats in the vehicle group and 9-PH group 24 h, 72 h and 168 h after TBI (*n* = 6). *p<0.05, **p<0.01, and ***p<0.001.

The chemical structure of the specific TRPM4 inhibitor is shown in Figure 1A. The brain water content of the ipsilateral hemisphere 24 h after TBI was measured to reflect the degree of cerebral edema (CE). Undoubtedly, the results showed that rats who suffered traumatic brain injury had a higher brain water content than rats who underwent sham operation. However, 9-PH treatment reduced the degree of edema at 24 h after TBI compared with vehicle treatment (Figure 1B). Correspondingly, the behavior assessment results in Figures 1C, D show that rats exhibited fewer functional deficits in the TBI+9-PH group than in the TBI + vehicle group when assessed by the modified Garcia test and the turning test. Thus, our findings indicated that 9-PH has the potential to alleviate CE after TBI.

### 9-Phenanthrol decreased rat serum SUR1 and TRPM4 levels after TBI

Previous studies have reported that proteins in brain tissue could be secreted into blood through the disrupted blood‒brain barrier (BBB) (Jha et al., 2017; Dundar et al., 2020). Therefore, in this experiment, rat serum SUR1 and TRPM4 levels were measured using ELISA 0 h, 6 h, 24 h, 48 h, 72 h, 120 h and 168 h after injury, and a diagram of the trends of change in the serum SUR1 and TRPM4 levels was drawn. As shown in Figure 1E, the concentrations of serum SUR1 and TRPM4 only slightly increased within 24 h after injury, but both of them significantly increased and peaked at Day 2 and Day 3 and then gradually decreased to the baseline level (0 h after injury) until the 7th day in the rats in the TBI + non-intervention group. Subsequently, the serum SUR1 and TRPM4 levels of the vehicle-treated and 9-PH-treated rats were determined on the 1st, 3rd and 7th days after injury to clarify whether 9-PH treatment has an effect on serum SUR1 and TRPM4 levels. As shown in Figures 1F, G, after treatment with 9-PH, the rats had lower serum SUR1 and serum TRPM4 levels than the rats treated with vehicle on the 3rd day after injury, but there was no obvious significant difference in the two indices between the two groups on the 1st day and 7th day after TBI. In summary, our results indicate that serum SUR1 and TRPM4 levels were increased and reached a single peak after TBI, and 9-ph treatment had a significant effect on reducing the peak of serum SUR1 and TRPM4 levels.

### 9-Phenanthrol attenuated hemorrhage and the fragmentation of capillaries

**FIGURE 2:**
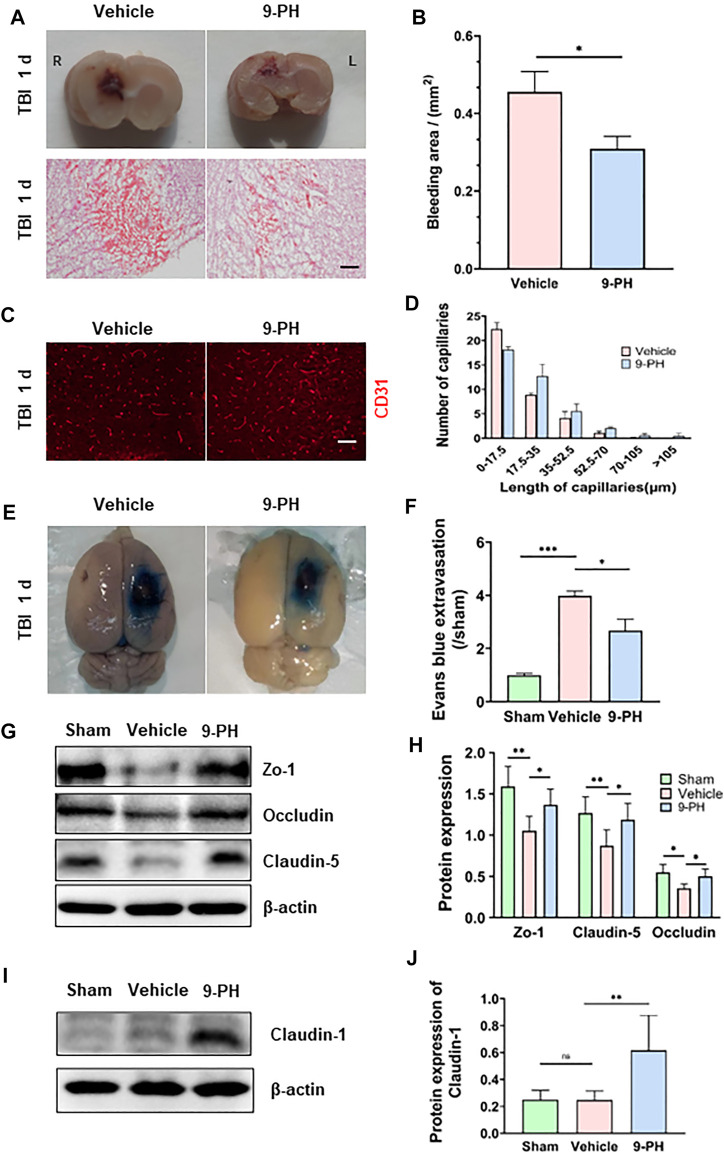
Protective effects of 9-PH on capillaries and the blood‒brain barrier. **(A)** Representative coronal brain specimens (upper) and hematoxylin and eosin (H&E) staining (bottom) of the bleeding area 24 h after injury. Red patches indicate hemorrhage. Scale bar: 100 μm. **(B)** Quantitative analysis of bleeding areas by H&E staining (*n* = 3). **(C, D)** Representative immunofluorescence images and quantitative analysis of capillaries (CD31) in the surrounding tissues 24 h after TBI (*n* = 3). Scale bar: 50 μm. **(E, F)** Evans blue extravasation of the ipsilateral brain was measured 24 h after TBI (*n* = 3). **(G, H)** Western blotting and quantitative analysis of the expression of the blood‒brain barrier tight junction proteins ZO-1, Occludin and Claudin-5 at 24 h after TBI in peripheral tissues (*n* = 5). **(I, J)** Western blotting and quantitative analysis of the expression of Claudin-1 24 h after TBI in peripheral tissues (*n* = 5). *p<0.05, **p<0.01, and ***p<0.001.

At 24 h after TBI, the injury size was significantly smaller in rats in the 9-PH group than in those in the vehicle group. Then, H&E staining was performed to assess the amount of hemorrhage. The results showed that the degree of bleeding was also significantly different between the 9-PH group and Vehicle group. The bleeding area of the brains in the 9-PH-treated group was smaller than that in the vehicle-treated group (Figures 2A, B). Additionally, we analyzed capillaries near the injury center, and the CD31 immunostaining results showed that there were many broken fragments of capillaries around the injury centers in the vehicle group, whereas 9-PH treatment significantly increased the capillary length and reduced capillary fragmentation (Figures 2C, D), which, to some extent, explains why 9-PH could reduce hemorrhage after TBI.

### 9-Phenanthrol protected the integrity of the blood‒brain barrier

The blood‒brain barrier consists of endothelial cells with tight junctions, a basement membrane and astrocyte foot processes. The above results indicate the protective effect of 9-PH on capillaries. Next, we further assessed the effects of 9-PH on blood‒brain barrier protection by Evans blue permeability assessment and molecular analysis of tight junctions of the blood‒brain barrier. The extravasation of Evans Blue in rats that underwent 9-PH treatment was remarkably lower than that of rats in the vehicle group 24 h after TBI (Figures 2E, F). As the Western blotting results showed, the expression of the tight junction proteins Zo-1, Occludin and Claudin-5 was also significantly reduced in tissues at 24 h after TBI. As expected, 9-PH treatment significantly reduced the loss of Zo-1, Occludin and Claudin5 (Figures 2G, H). To verify whether 9-PH has positive effects on promoting BBB repair, we quantified the expression of Claudin-1, which is considered a marker for acute repair of the BBB (Chen et al., 2019b). Consistent with previous studies, there was almost no expression of Claudin1 in the Sham group and only slight expression of Claudin1 in the vehicle group. Similar to our hypothesis, Claudin-1 expression was significantly upregulated in the 9-PH treatment group (Figures 2I, J). These results demonstrate that 9-PH plays a protective role in BBB integrity partly by inhibiting the loss of tight junction proteins and promoting Claudin-1 upregulation after brain injury.

### 9-Phenanthrol inhibited apoptosis and promoted neuronal survival after TBI

**FIGURE 3:**
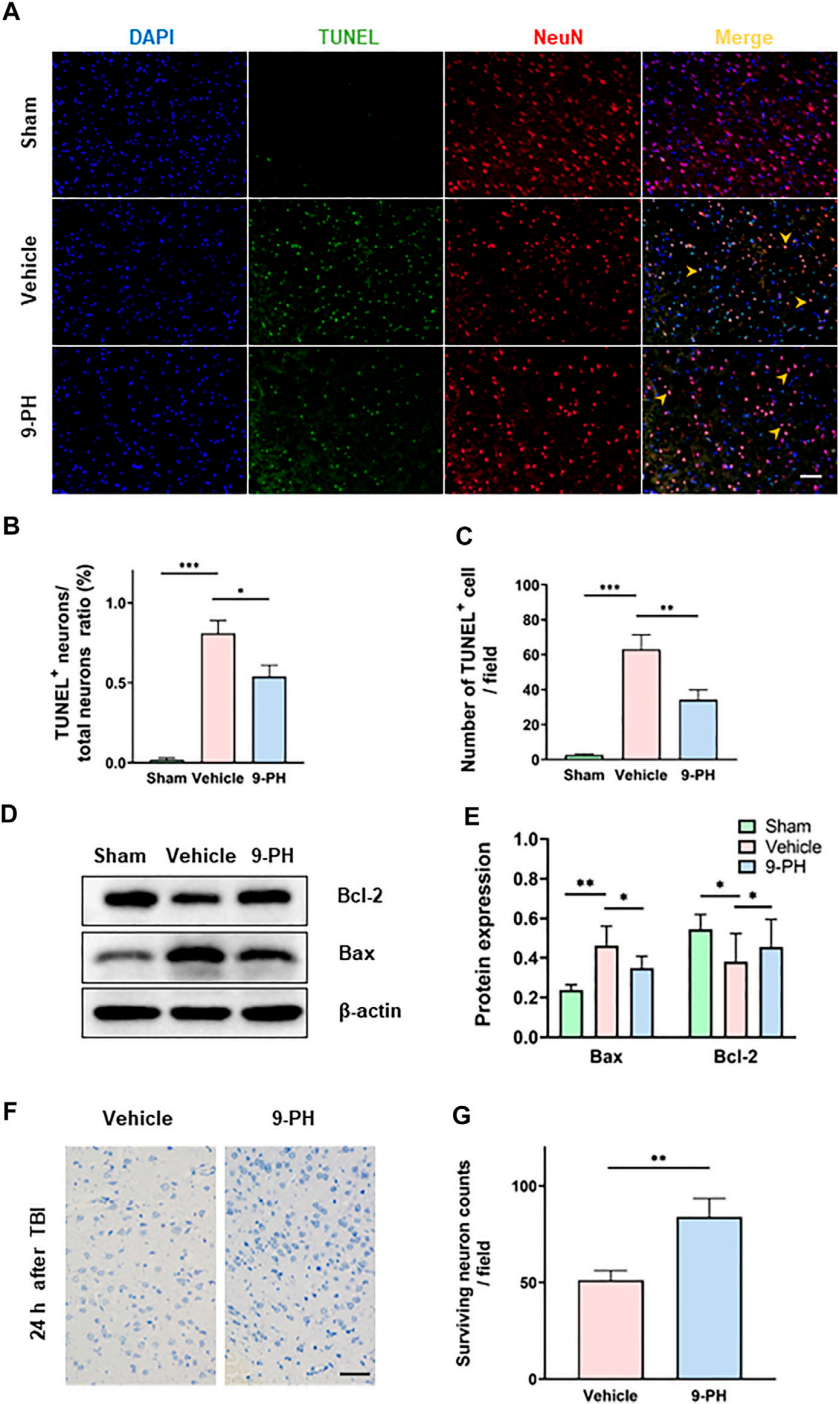
9-PH reduces cell apoptosis and promotes neuronal survival after TBI. **(A)** Images of TUNEL (green) and NeuN (red) double immunostaining 24 h after TBI. DAPI (blue) represents nuclei, and yellow arrows indicate apoptotic neurons in MERGE images. Scale bar: 50 μm. **(B, C)** Quantitative analysis of the apoptotic neuron/neuron ratio and apoptotic cell counts (*n* = 3). **(D, E)** Protein expression determination and quantitative analysis of Bcl2 and Bax in surrounding tissue at 24 h after TBI (*n* = 5). **(F, G)** Representative NISSL staining images and quantitative analysis of surviving neurons at 24 h after injury (*n* = 3). Scale bar: 20 μm *p < 0.05, **p < 0.01, and ***p < 0.001.

The imbalance of intracellular and extracellular ions and blood leakage caused by the destruction of the BBB are related to neuronal death (Jha et al., 2019). Double immunofluorescence of NeuN-labeled neurons and TUNEL-stained apoptotic cells was performed to detect neuronal apoptosis, and the percentage of TUNEL-positive neurons to total neurons in the 9-PH group was lower than that in the vehicle group at 24 h after TBI. In addition, the number of TUNEL-positive cells was also decreased in the 9-PH-treated group (Figures 3A–C). Next, we measured the expression f apoptosis-related proteins (Bax and Bcl2) in tissues around the injury 24 h after TBI. Encouragingly, 9-PH reduced the expression of the apoptosis-promoting factor Bax and increased the expression of the apoptosis-inhibiting factor Bcl-2 24 h after TBI (Figures 3D, E). As represented in Figure 3F, neurons in the 9-PH group exhibited clearer cell contours and deeper staining, which indicated less damage according to the Nissl staining of tissues near the injury center 24 h after TBI. Quantitative analysis further demonstrated that the number of surviving neurons was significantly higher in rats that underwent 9-PH treatment after TBI than in rats that underwent vehicle treatment (Figure 3G).

### 9-Phenanthrol reduced the proliferation of microglia and suppressed astrogliosis

**FIGURE 4:**
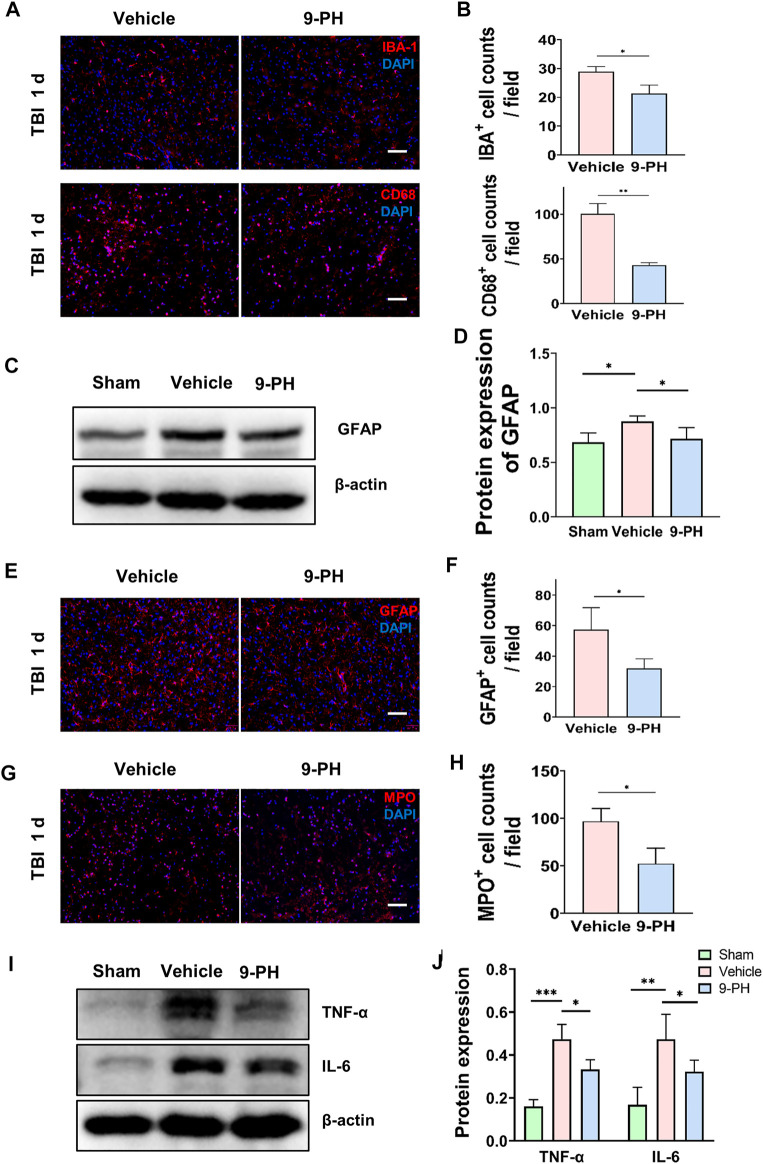
Inhibitory effect of 9-PH on the inflammatory response after TBI. **(A, B)** Representative immunofluorescence images and quantitative analysis of IBA-1- and CD68-positive cells in surrounding tissue at 24 h after TBI (*n* = 3). Nuclei are stained with DAPI (blue). Scale bars: 50 μm. **(C, D)** Western blotting and quantitative analysis of GFAP expression in surrounding tissue 24 h after TBI (*n* = 5). **(E, F)** Images of GFAP immunostaining and quantitative analysis of GFAP-positive astrocytes (*n* = 3). DAPI (blue) was used to label nuclei. Scale bar: 50 μm. **(G, H)** Immunofluorescence staining with MPO and quantitative analysis of infiltrated neutrophils (MPO positive) in surrounding tissue at 24 h after TBI (*n* = 3). DAPI (blue) represents nuclei. Scale bars: 50 μm. **(I, J)** Western blotting and quantitative analysis of TNF-α and IL-6 in surrounding tissue 24 h after TBI (*n* = 5). *p<0.05, **p<0.01, and ***p<0.001.

Microglia are important immune cells in the central nervous system. A previous study reported that the activation and proliferation of microglia is related to BBB destruction and neuronal loss (Liao et al., 2020; Chen et al., 2022; He et al., 2022). Twenty-4 hours after injury, immunofluorescence staining for IBA-1 showed that 9-PH treatment reduced the number of IBA-1-positive microglia. Notably, there appeared to be fewer amoeboid microglia with larger bodies and fewer branches, which are considered activated microglia, in the 9-PH group. CD68, which is mainly expressed in activated microglia/macrophages but not resting microglia, was also sequentially labeled by immunostaining. As the results show, there were fewer CD68-positive cells in the 9-PH group than in the vehicle group (Figures 4A, B). Increased expression of glial fibrillary acidic protein (GFAP) is the basis of activation and proliferation of astrocytes, which is often considered a unique response of the central nervous system (CNS) to many injuries. We then sought to determine whether astrogliosis was reduced following inhibition of microglial activation with 9-PH. Quantitative analysis of protein showed that 9-PH also reduced the expression of GFAP 24 h after TBI (Figures 4C, D). Further immunofluorescence analysis of GFAP showed that the number of GFAP-positive astrocytes increased 24 h after TBI in the 9-PH group compared to the vehicle group (Figures 4E, F). These results indicated that 9-PH may also inhibit the proliferation of activated microglia and astrocytes to reduce secondary injury after TBI.

### 9-Phenanthrol alleviated neutrophil infiltration and inflammatory factor release

Because of the existence of the blood‒brain barrier, the brain is protected from damage to immune cells and inflammatory factors originating from the peripheral blood. After CNS injury, inflammation can aggravate the destruction of the blood‒brain barrier (Yang et al., 2019), and peripheral immune cells can enter brain tissue through the disrupted BBB to regulate the inflammatory reaction. Therefore, in this experiment, we analyzed neutrophil infiltration in the brain. Immunostaining for MPO was used to label neutrophils 24 h after TBI. As shown in Figures 4G, H, a large number of MPO-positive neutrophils infiltrated into the brain tissue of rats in the vehicle group after injury, and this infiltration of neutrophils was inhibited by treatment with 9-PH (Figures 4G, H). Since TNF-α and IL-6 are two critical proinflammatory factors secreted by resident microglia and peripheral neutrophils that migrate to the site of injury after TBI (Chen et al., 2018), we chose TNF-α and IL-6 as the representative markers of inflammation and used Western blotting to measure their expression. The results showed that 9-PH treatment also reduced the expression of the inflammatory mediators TNF-α and IL-6 in brain tissue at 24 h after TBI (Figures 4I, J).

### 9-Phenanthrol inhibited the overexpression of TRPM4 and MMP-9

**FIGURE 5:**
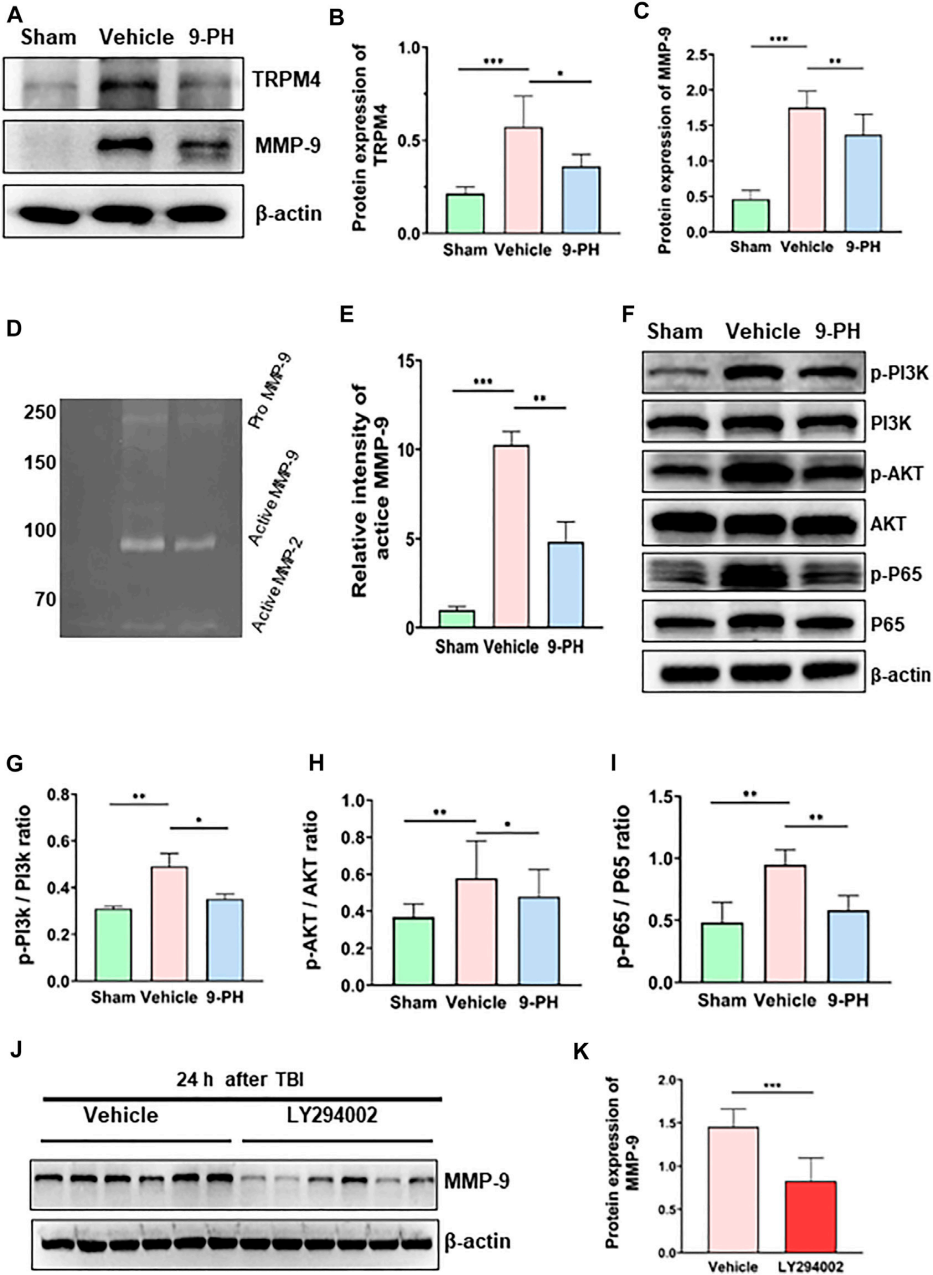
9-PH inhibits the expression of TRPM4 and MMP-9 expression after TBI. **(A–C)** Representative protein expression results and densitometric analysis of TRPM4 and MMP-9 in the surrounding tissue 24 h after TBI (*n* = 5). **(D, E)** Gelatin zymography and densitometric analysis of zymography in the surrounding tissue 24 h after TBI (*n* = 3). **(F–I)** Quantitative determination of protein and ratio analysis of phosphorylated-PI3K/PI3K, phosphorylated-AKT/AKT, and phosphorylated-P65/P65 24 h after TBI (*n* = 5). **(J, K)** Western blotting and densitometric analysis of MMP-9 expression in the LY294002 group and Vehicle group 24 h after TBI (*n* = 6). *p<0.05, **p<0.01, and ***p<0.001.

Because TRMP4 is the target of 9-PH, the levels of TRPM4 were tested to determine whether 9-PH affects the expression of TRPM4. The results showed that the expression of TRPM4 in the Vehicle group was significantly higher than that in the Sham group, and 9-PH treatment also significantly reduced the protein levels of TRPM4 in surrounding tissues at 24 h after TBI (Figures 5A, B). This result suggests that in addition to inhibiting the function of TRPM4, 9-PH treatment may also inhibit the expression of TRPM4. MMP-9 has been proven to have strong activity in degrading tight junction proteins of the BBB (Chaturvedi and Kaczmarek, 2014). The results showed almost no expression of MMP-9 in the Sham group. At 24 h after injury, the expression level of MMP-9 in the Vehicle group increased dramatically, and 9-PH treatment significantly reduced the expression of MMP-9 (Figures 5A, C). Moreover, through the gelatin zymogram results, we found that the enzyme activity of MMP-9 in the 9-PH treatment group was significantly lower than that in the Vehicle group 24 h after injury (Figures 5D, E).

### 9-Phenanthrol downregulated the expression of MMP-9 by inhibiting the PI3K/AKT/NF-kB signaling pathway

Previous studies have shown that activation of the PI3K/AKT/NF-kB signaling pathway participates in promoting MMP-9 expression in many tissues (Dilly et al., 2013; Ha et al., 2019; Qin et al., 2019). To explore the mechanism by which 9-PH inhibits the upregulation of MMP-9 after TBI, we measured the activation of this pathway. Western blotting results showed increased expression of phosphorylated PI3K-p85, phosphorylated AKT-p55 and phosphorylated NF-kB-p65 in brain tissue at 24 h after injury compared with the Sham group, while the total expression of PI3K-p85, AKT-p55 and NF-kB-p65 remains unchanged. After 9-PH treatment, the expression of phosphorylated PI3K-p85, phosphorylated AKT-p55 and phosphorylated NF-kB-p65 and the ratios of p-PI3K-p85/PI3K-p85, p-AKT-p55/AKT-p55 and p-NF-kB-p65/NF-kB-p65 were all decreased (Figures 5F–I). The results showed that the PI3K/AKT/NF-kB signaling pathway was activated after TBI and 9-PH could block this signaling pathway by inhibiting the phosphorylation of PI3K, AKT and NF-kB. Furthermore, we used the PI3K-specific inhibitor LY294002 to interfere with this pathway to determine whether this pathway is related to the expression of MMP-9. As a result, blocking of this pathway by LY294002 significantly reduced the expression of MMP-9 at 24 h after TBI (Figures 5J, K). Based on these results, we consider that 9-PH inhibits the expression of MMP-9 at least partially by blocking the activation of the PI3K/AKT/NF-kB signaling pathway after TBI.

## Discussion

At present, there is a consensus that cerebral edema (CE) caused by the disruption of endothelial cells (ECs) and the blood‒brain barrier (BBB) is an important basic pathological process of secondary brain injury and induces poor prognosis. Here, we established a rat TBI model to study the effects of 9-PH on alleviating CE. The results of this study showed that 9-PH treatment can significantly reduce brain water content and promote neurological deficits after TBI. 9-PH significantly reduced the fragmentation of capillaries and disruption of the BBB around the injured tissues and reduced Evans blue extravasation. In addition, our results indicate that 9-PH may inhibit the expression of MMP-9 by inhibiting the activation of the PI3K/AKT/NF-kB pathway, which implies that TRPM4 may activate this pathway to directly promote MMP-9 expression after TBI.

CE is mainly divided into two categories: cytotoxic edema and vasogenic edema (Klatzo, 1967). Opening of the TRPM4 channel on the cell membrane mediates the influx of sodium ions, which is accompanied by a large amount of water entering the cell *via* aquaporin 4 (AQP4), leading to cell swelling and disrupted energy metabolism, and producing many toxic substances, and eventually leading to the death of microvascular unit cells, including ECs; this is currently considered the main mechanism underlying TRPM4-mediated edema formation (Stokum et al., 2018). Consistent with the previous experimental results of SUR1-TRPM4 inhibition, our studies demonstrate that 9-PH significantly reduced CE and neurological impairment in rats after TBI (Jiang et al., 2017; Chen et al., 2019b). In our experiment, 9-PH treatment significantly inhibited the upregulation of TRPM4. However, there is no research evidence suggesting that 9-PH directly inhibits the expression of TRPM4. We speculate that one of the reasons is that CE inhibition by 9-PH alleviates various secondary injuries mediated by TRPM4 and then weakens the stimulation of TRPM4 expression. Similar results were also observed in an experiment in which spinal cord injury (SCI) was treated with FFA. The anti-inflammatory effect and partial inhibitory effect of FFA on TRPM4 also attenuated the *de novo* expression of TRPM4 induced by SCI (Chen et al., 2022). The specific reasons for this amazing phenomenon need to be further elucidated.

The blood‒brain barrier plays an important role in maintaining the function of the central nervous system (CNS). Damage to BBB integrity can cause macromolecules to leak from the blood into the extracellular space of the brain, which eventually results in processive water accumulation and vascular edema. Our research has shown that 9-PH can reduce Evans blue extravasation, indicating that BBB integrity has been improved. 9-PH can inhibit the loss of tight junction proteins (Zo-1, Occludin, Claudin-5), and9-PH treatment promotes the expression of Claudin-1, which is considered to play an important role in the repair of the BBB in the acute stage after injury (Chen et al., 2019b). This shows that 9-PH also has effects on the acute repair of the BBB. Moreover, our immunofluorescence results show that 9-PH is also of great significance in reducing endothelial cell damage and reducing vascular fragmentation, which can decrease hemorrhage and thus alleviate edema and secondary injuries (Griffiths et al., 1978; Simard et al., 2007). All these results show the benefits of 9-PH in inhibiting vasogenic CE. Additionally, 9-PH also promotes neuronal survival and inhibits neuronal apoptosis after TBI.

A main reason that 9-PH reduces BBB damage is that 9-PH can inhibit the upregulation and enzymatic activity of MMP-9. MMP-9 is a member of the metalloproteinase (MMP) family and is involved in the secondary injury process after CNS injury (Chaturvedi and Kaczmarek, 2014). It can degrade tight junction proteins and the basement membrane of the BBB and destroy the integrity of the blood‒brain barrier structure (Turner and Sharp, 2016). Increasing evidence indicates that TRPM4 may participate in the expression of MMP-9 to accelerate the development of angiogenic edema (Lee et al., 2014; Jiang et al., 2017; Gerzanich et al., 2018). We found that 9-PH significantly inhibited the activation of the PI3K/AKT/NF-kB signaling pathway, which has been reported to be involved in the expression of MMP-9 in many diseases (Dilly et al., 2013; Qin et al., 2019; Peng et al., 2020). Later, after targeting this pathway with the PI3K inhibitor LY294002, we found that inhibition of this pathway significantly reduced the expression of MMP-9. Therefore, we believe that TRPM4 may promote the regulation of MMP-9 secretion through the PI3K/AKT/NF-kB signaling pathway, thereby further aggravating BBB damage, and that 9-PH can block this process. Interestingly, a previous study reported that the intervention of SUR1-TRPM4 with GLC did not improve CE after collagenase-induced intracerebral hemorrhage (Wilkinson et al., 2019; Kung et al., 2021). Our explanation for this result is that the destruction of the BBB is comediated by MMP-9 and collagenase in the acute phase (within the first 24 h) of collagenase-induced intracerebral hemorrhage in rats. Although GLC can reduce the expression and activity of MMP-9 by inhibiting the SUR1-TRPM4 complex, it cannot inhibit the destruction of the BBB induced by collagenase. These results suggest that angiogenic CE plays a very important role in SUR1-TRPM4-mediated CE. Another implication is that GLC may be insufficient in inhibiting the expression and activity of MMP-9.

Several clinical studies suggest that serum SUR1 and TRPM4 concentrations change with disease progression after CNS injury because of disruption of the BBB, suggesting that serum SUR1 and serum TRPM4 levels may have the potential to be new markers of CNS injury to reflect disease progression (Jha et al., 2017; Dundar et al., 2020; Zhuge et al., 2022). In this study, we found that the serum SUR1 and TRPM4 concentrations showed a single peak change in the acute phase in rats after TBI. Interestingly, 9-PH treatment can significantly reduce these peak levels in rat serum, which further enhances the potential of serum SUR1 and TRPM4 to be biomarkers in CNS diseases.

Our work also shows the effects of 9-PH on inhibiting the inflammatory response. Microglia are the most important inflammatory cells in the CNS. Activated microglia can also damage normal cells and release more signals to expand the inflammatory response while participating in the immune response. In addition, activated microglia also participate in the activation of astrocytes, which can further increase tissue damage (Popovich et al., 1997; Emmetsberger and Tsirka, 2012). Some new research results have shown that SUR1-TRPM4 may not participate in inflammatory reactions by reducing tissue swelling. For example, inhibiting SUR1-TRPM4 can reduce the release of inflammatory mediators in tissue without obvious edema. Furthermore, SUR1-TRPM4 was proven to promote the activation of NLRP3 and TLR4 in microglia (Makar et al., 2015; Kurland et al., 2016; He et al., 2022). After 9-PH treatment, the number of activated microglia/macrophages and astrocytes and the number of infiltrating neutrophils in the brain tissue of rats all significantly decreased, and the inflammatory mediators TNF-α and IL-6 were expressed at lower levels. Neutrophil infiltration may be related to the integrity of the BBB (Taoka et al., 1997; Saiwai et al., 2010). This is a benign cycle: the reduction in inflammatory reactions reduces the destruction of the BBB, and a less injured BBB can reduce the toxic effects of substances from the blood on brain tissue.

To date, GLC and the monoclonal antibody M4P are the most widely studied drugs that target SUR1-TRPM4 for the treatment of CE. Although recent achievements are encouraging, studies on SUR1 or TRPM4 still have the following problems: ① The time window for treatment is limited, and only the treatments for early CE has been studied. ② The inhibition of Na + influx by GLC only promotes the redistribution of water in brain tissue, but the reduction of CE and intracranial hypertension is limited. ③ Inhibition of GLC to SUR1-TRPM4 channel depends on the coexpression of SUR1 and TRPM4 in a certain ratio, and GLC has no or limited effect on those TRPM4 channels which do not assemble with SUR1. ④ The routine dose of GLC is low, so the signs of CE remission are not obvious, while a high dose leads to continuous hypoglycemia, which has potential risks. ⑤ Monoclonal antibodies targeting TRPM4 have difficulty penetrating the BBB due to their large molecular weight, and their clinical application is limited by immunogenic factors. ⑥ The inhibition of MMP-9 secretion by GLC is not sufficiently effective. Therefore, it is still a challenge to further clarify the mechanisms underlying these events and develop effective blockers that target TRPM4.

Based on the results of this study, 9-PH has significant advantages in exerting neuroprotective effects after experimental TBI. On the one hand, 9-PH reduces cytotoxic edema by inhibiting the expression and function of TRPM4; on the other hand, it reduces the destruction of the BBB and vasogenic edema by reducing the expression and activity of MMP-9 and promoting the expression of Claudin-1. Compared with GLC, 9-PH is more specific in inhibiting TRPM4 and does not depend on the coassembly of SUR1-TRPM4. Moreover, 9-PH is more effective in inhibiting the activity of MMP-9, and it can restore decreased cerebral blood flow after injury (Gong et al., 2019). In general, we think 9-PH has the potential to be a new CE treatment and has important value in treating CE alone or together with GLC in some specific situations (such as patients with hypoglycemia). In view of its smaller molecular weight, 9-PH may easily cross the blood‒brain barrier where tight junctions have not been completely disrupted, further effectively enhancing cell survival and even preventing expansion of injured areas; thus, it is more suitable for early treatment or prevention of progressive diseases or minor injuries. Since this study only discussed the effects of 9-PH in the acute phase and did not directly compare the effects of 9-PH and GLC, more studies are needed to explore the effects of 9-PH. Moreover, the concentrations of serum SUR1 and TRPM4 can reflect the course of TBI in rats, so they may have value for disease prediction and can be considered potential biological markers.

## Data Availability

The original contributions presented in the study are included in the article/supplementary material, further inquiries can be directed to the corresponding authors.

## References

[B1] Adukauskiene D. Bivainyte A. Radaviciute E. (2007). Cerebral edema and its treatment. Med. Kaunas. 43 (2), 170–176. 10.3390/medicina43020021 17329953

[B2] Altay O. Hasegawa Y. Sherchan P. Suzuki H. Khatibi N. H. Tang J. (2012). Isoflurane delays the development of early brain injury after subarachnoid hemorrhage through sphingosine-related pathway activation in mice. Crit. Care Med. 40 (6), 1908–1913. 10.1097/CCM.0b013e3182474bc1 22488000 PMC3358576

[B3] Amarouch M. Y. Syam N. Abriel H. (2013). Biochemical, single-channel, whole-cell patch clamp, and pharmacological analyses of endogenous TRPM4 channels in HEK293 cells. Neurosci. Lett. 541, 105–110. 10.1016/j.neulet.2013.02.011 23428507

[B4] Chaturvedi M. Kaczmarek L. (2014). Mmp-9 inhibition: A therapeutic strategy in ischemic stroke. Mol. Neurobiol. 49 (1), 563–573. 10.1007/s12035-013-8538-z 24026771 PMC3918117

[B5] Chen B. Gao Y. Wei S. Low S. W. Ng G. Yu D. (2019a). TRPM4-specific blocking antibody attenuates reperfusion injury in a rat model of stroke. Pflugers Arch. 471 (11-12), 1455–1466. 10.1007/s00424-019-02326-8 31664513 PMC6892354

[B6] Chen B. Ng G. Gao Y. Low S. W. Sandanaraj E. Ramasamy B. (2019b). Non-invasive multimodality imaging directly shows TRPM4 inhibition ameliorates stroke reperfusion injury. Transl. Stroke Res. 10 (1), 91–103. 10.1007/s12975-018-0621-3 29569041 PMC6327008

[B7] Chen J. Chang Y. Zhu J. Peng Y. Li Z. Zhang K. (2022). Flufenamic acid improves survival and neurologic outcome after successful cardiopulmonary resuscitation in mice. J. Neuroinflammation 19 (1), 214. 10.1186/s12974-022-02571-2 36050694 PMC9438280

[B8] Chen S. Peng J. Sherchan P. Ma Y. Xiang S. Yan F. (2020). TREM2 activation attenuates neuroinflammation and neuronal apoptosis via PI3K/Akt pathway after intracerebral hemorrhage in mice. J. Neuroinflammation 17 (1), 168. 10.1186/s12974-020-01853-x 32466767 PMC7257134

[B9] Chen S. Shao L. Ma L. (2021). Cerebral edema formation after stroke: Emphasis on blood-brain barrier and the lymphatic drainage system of the brain. Front. Cell Neurosci. 15, 716825. 10.3389/fncel.2021.716825 34483842 PMC8415457

[B10] Chen X. Chen C. Fan S. Wu S. Yang F. Fang Z. (2018). Omega-3 polyunsaturated fatty acid attenuates the inflammatory response by modulating microglia polarization through SIRT1-mediated deacetylation of the HMGB1/NF-κB pathway following experimental traumatic brain injury. J. Neuroinflammation 15 (1), 116. 10.1186/s12974-018-1151-3 29678169 PMC5909267

[B11] Dilly A. K. Ekambaram P. Guo Y. Cai Y. Tucker S. C. Fridman R. (2013). Platelet-type 12-lipoxygenase induces MMP9 expression and cellular invasion via activation of PI3K/Akt/NF-κB. Int. J. Cancer 133 (8), 1784–1791. 10.1002/ijc.28165 23526143 PMC4269488

[B12] Dundar T. T. Abdallah A. Yurtsever I. Guler E. M. Ozer O. F. Uysal O. (2020). Serum SUR1 and TRPM4 in patients with subarachnoid hemorrhage. Neurosurg. Rev. 43 (6), 1595–1603. 10.1007/s10143-019-01200-6 31707576

[B13] Emmetsberger J. Tsirka S. E. (2012). Microglial inhibitory factor (MIF/TKP) mitigates secondary damage following spinal cord injury. Neurobiol. Dis. 47 (3), 295–309. 10.1016/j.nbd.2012.05.001 22613732 PMC3876729

[B14] Faden A. I. (2002). Neuroprotection and traumatic brain injury: Theoretical option or realistic proposition. Curr. Opin. Neurol. 15 (6), 707–712. 10.1097/01.wco.0000044767.39452.bf 12447109

[B15] Feeney D. M. Boyeson M. G. Linn R. T. Murray H. M. Dail W. G. (1981). Responses to cortical injury: I. Methodology and local effects of contusions in the rat. Brain Res. 211 (1), 67–77. 10.1016/0006-8993(81)90067-6 7225844

[B16] Fu S. Luo X. Wu X. Zhang T. Gu L. Wang Y. (2020). Activation of the melanocortin-1 receptor by NDP-MSH attenuates oxidative stress and neuronal apoptosis through PI3K/Akt/Nrf2 pathway after intracerebral hemorrhage in mice. Oxid. Med. Cell. Longev. 2020, 8864100. 10.1155/2020/8864100 33274009 PMC7676969

[B17] Gerzanich V. Kwon M. S. Woo S. K. Ivanov A. Simard J. M. (2018). SUR1-TRPM4 channel activation and phasic secretion of MMP-9 induced by tPA in brain endothelial cells. PLoS ONE 13 (4), e0195526. 10.1371/journal.pone.0195526 29617457 PMC5884564

[B18] Gong Y. Du M.-Y. Yu H.-L. Yang Z.-Y. Li Y.-J. Zhou L. (2019). Increased TRPM4 activity in cerebral artery myocytes contributes to cerebral blood flow reduction after subarachnoid hemorrhage in rats. Neurotherapeutics 16 (3), 901–911. 10.1007/s13311-019-00741-4 31073979 PMC6694375

[B19] Grand T. Demion M. Norez C. Mettey Y. Launay P. Becq F. (2008). 9-phenanthrol inhibits human TRPM4 but not TRPM5 cationic channels. Br. J. Pharmacol. 153 (8), 1697–1705. 10.1038/bjp.2008.38 18297105 PMC2438271

[B20] Griffiths I. R. Burns N. Crawford A. R. (1978). Early vascular changes in the spinal grey matter following impact injury. Acta Neuropathol. 41 (1), 33–39. 10.1007/BF00689554 636835

[B21] Guinamard R. Hof T. Del Negro C. A. (2014). The TRPM4 channel inhibitor 9-phenanthrol. Br. J. Pharmacol. 171 (7), 1600–1613. 10.1111/bph.12582 24433510 PMC3966741

[B22] Guinamard R. Salle L. Simard C. (2011). The non-selective monovalent cationic channels TRPM4 and TRPM5. Adv. Exp. Med. Biol. 704, 147–171. 10.1007/978-94-007-0265-3_8 21290294

[B23] Ha S. H. Kwon K. M. Park J. Y. Abekura F. Lee Y. C. Chung T. W. (2019). Esculentoside H inhibits colon cancer cell migration and growth through suppression of MMP-9 gene expression via NF-kB signaling pathway. J. Cell. Biochem. 120 (6), 9810–9819. 10.1002/jcb.28261 30525244

[B24] Hackenberg K. Unterberg A. (2016). Traumatic brain injury. Nervenarzt 87 (2), 203–214. 10.1007/s00115-015-0051-3 26810405

[B25] He Y. Chang Y. Peng Y. Zhu J. Liu K. Chen J. (2022). Glibenclamide directly prevents neuroinflammation by targeting SUR1-TRPM4-mediated NLRP3 inflammasome activation in microglia. Mol. Neurobiol. 59 (10), 6590–6607. 10.1007/s12035-022-02998-x 35972671

[B26] Huttner H. B. Schwab S. (2009). Malignant middle cerebral artery infarction: Clinical characteristics, treatment strategies, and future perspectives. Lancet Neurol. 8 (10), 949–958. 10.1016/S1474-4422(09)70224-8 19747656

[B27] Jassam Y. N. Izzy S. Whalen M. McGavern D. B. El Khoury J. (2017). Neuroimmunology of traumatic brain injury: Time for a paradigm shift. Neuron 95 (6), 1246–1265. 10.1016/j.neuron.2017.07.010 28910616 PMC5678753

[B28] Jha R. M. Kochanek P. M. Simard J. M. (2019). Pathophysiology and treatment of cerebral edema in traumatic brain injury. Neuropharmacology 145, 230–246. 10.1016/j.neuropharm.2018.08.004 30086289 PMC6309515

[B29] Jha R. M. Puccio A. M. Chou S. H. Chang C. H. Wallisch J. S. Molyneaux B. J. (2017). Sulfonylurea receptor-1: A novel biomarker for cerebral edema in severe traumatic brain injury. Crit. Care Med. 45 (3), e255–e264. 10.1097/CCM.0000000000002079 27845954 PMC5550829

[B30] Jiang B. Li L. Chen Q. Tao Y. Yang L. Zhang B. (2017). Role of glibenclamide in brain injury after intracerebral hemorrhage. Transl. Stroke Res. 8 (2), 183–193. 10.1007/s12975-016-0506-2 27807801

[B31] King Z. A. Sheth K. N. Kimberly W. T. Simard J. M. (2018). Profile of intravenous glyburide for the prevention of cerebral edema following large hemispheric infarction: Evidence to date. Drug Des. devel. Ther. 12, 2539–2552. 10.2147/DDDT.S150043 PMC6101021 30147301

[B32] Klatzo I. (1967). Presidental address. Neuropathological aspects of brain edema. J. Neuropathol. Exp. Neurol. 26 (1), 1–14. 10.1097/00005072-196701000-00001 5336776

[B33] Kung T. F. C. Wilkinson C. M. Dirks C. A. Jickling G. C. Colbourne F. (2021). Glibenclamide does not improve outcome following severe collagenase-induced intracerebral hemorrhage in rats. PLoS ONE 16 (6), e0252584. 10.1371/journal.pone.0252584 34081746 PMC8174736

[B34] Kurland D. B. Gerzanich V. Karimy J. K. Woo S. K. Vennekens R. Freichel M. (2016). The Sur1-Trpm4 channel regulates NOS2 transcription in TLR4-activated microglia. J. Neuroinflammation 13 (1), 130. 10.1186/s12974-016-0599-2 27246103 PMC4888589

[B35] Lee J. Y. Choi H. Y. Na W. H. Ju B. G. Yune T. Y. (2014). Ghrelin inhibits BSCB disruption/hemorrhage by attenuating MMP-9 and SUR1/TrpM4 expression and activation after spinal cord injury. Biochim. Biophys. Acta 1842 (12), 2403–2412. 10.1016/j.bbadis.2014.09.006 25261791

[B36] Li G. Makar T. Gerzanich V. Kalakonda S. Ivanova S. Pereira E. F. R. (2020). HIV-1 vpr-induced proinflammatory response and apoptosis are mediated through the sur1-trpm4 channel in astrocytes. mBio 11 (6), e02939. 10.1128/mBio.02939-20 33293383 PMC8534293

[B37] Liao B. Geng L. Zhang F. Shu L. Wei L. Yeung P. K. K. (2020). Adipocyte fatty acid-binding protein exacerbates cerebral ischaemia injury by disrupting the blood-brain barrier. Eur. Heart J. 41 (33), 3169–3180. 10.1093/eurheartj/ehaa207 32350521 PMC7556749

[B38] Luo X. Li L. Zheng W. Gu L. Zhang X. Li Y. (2020). HLY78 protects blood-brain barrier integrity through Wnt/β-catenin signaling pathway following subarachnoid hemorrhage in rats. Brain Res. Bull. 162, 107–114. 10.1016/j.brainresbull.2020.06.003 32565130

[B39] Makar T. K. Gerzanich V. Nimmagadda V. K. Jain R. Lam K. Mubariz F. (2015). Silencing of Abcc8 or inhibition of newly upregulated Sur1-Trpm4 reduce inflammation and disease progression in experimental autoimmune encephalomyelitis. J. Neuroinflammation 12, 210. 10.1186/s12974-015-0432-3 26581714 PMC4652344

[B40] Mrejeru A. Wei A. Ramirez J. M. (2011). Calcium-activated non-selective cation currents are involved in generation of tonic and bursting activity in dopamine neurons of the substantia nigra pars compacta. J. Physiol. 589 (10), 2497–2514. 10.1113/jphysiol.2011.206631 21486760 PMC3115821

[B41] Peng X. Zhou J. Li B. Zhang T. Zuo Y. Gu X. (2020). Notch1 and PI3K/Akt signaling blockers DAPT and LY294002 coordinately inhibit metastasis of gastric cancer through mutual enhancement. Cancer Chemother. Pharmacol. 85 (2), 309–320. 10.1007/s00280-019-03990-4 31732769

[B42] Popovich P. G. Wei P. Stokes B. T. (1997). Cellular inflammatory response after spinal cord injury in Sprague-Dawley and Lewis rats. J. Comp. Neurol. 377 (3), 443–464. 10.1002/(sici)1096-9861(19970120)377:3<443:aid-cne10>3.0.co;2-s 8989657

[B43] Qin W. Li J. Zhu R. Gao S. Fan J. Xia M. (2019). Melatonin protects blood-brain barrier integrity and permeability by inhibiting matrix metalloproteinase-9 via the NOTCH3/NF-κB pathway. Aging 11 (23), 11391–11415. 10.18632/aging.102537 31811815 PMC6932927

[B44] Saiwai H. Ohkawa Y. Yamada H. Kumamaru H. Harada A. Okano H. (2010). The LTB4-BLT1 axis mediates neutrophil infiltration and secondary injury in experimental spinal cord injury. Am. J. Pathology 176 (5), 2352–2366. 10.2353/ajpath.2010.090839 PMC2861100 20304963

[B45] Sheth K. N. Elm J. J. Molyneaux B. J. Hinson H. Beslow L. A. Sze G. K. (2016). Safety and efficacy of intravenous glyburide on brain swelling after large hemispheric infarction (GAMES-RP): A randomised, double-blind, placebo-controlled phase 2 trial. Lancet Neurol. 15 (11), 1160–1169. 10.1016/S1474-4422(16)30196-X 27567243

[B46] Simard J. M. Tsymbalyuk O. Ivanov A. Ivanova S. Bhatta S. Geng Z. (2007). Endothelial sulfonylurea receptor 1-regulated NC Ca-ATP channels mediate progressive hemorrhagic necrosis following spinal cord injury. J. Clin. Invest. 117 (8), 2105–2113. 10.1172/JCI32041 17657312 PMC1924498

[B47] Stokum J. A. Kwon M. S. Woo S. K. Tsymbalyuk O. Vennekens R. Gerzanich V. (2018). SUR1-TRPM4 and AQP4 form a heteromultimeric complex that amplifies ion/water osmotic coupling and drives astrocyte swelling. Glia 66 (1), 108–125. 10.1002/glia.23231 28906027 PMC5759053

[B48] Taoka Y. Okajima K. Uchiba M. Murakami K. Kushimoto S. Johno M. (1997). Role of neutrophils in spinal cord injury in the rat. Neuroscience 79 (4), 1177–1182. 10.1016/s0306-4522(97)00011-0 9219976

[B49] Turner R. J. Sharp F. R. (2016). Implications of MMP9 for blood brain barrier disruption and hemorrhagic transformation following ischemic stroke. Front. Cell Neurosci. 10, 56. 10.3389/fncel.2016.00056 26973468 PMC4777722

[B50] Vennekens R. Nilius B. (2007). Insights into TRPM4 function, regulation and physiological role. Handb. Exp. Pharmacol. 179, 269–285. 10.1007/978-3-540-34891-7_16 17217063

[B51] Wilkinson C. M. Brar P. S. Balay C. J. Colbourne F. (2019). Glibenclamide, a Sur1-Trpm4 antagonist, does not improve outcome after collagenase-induced intracerebral hemorrhage. PLoS ONE 14 (5), e0215952. 10.1371/journal.pone.0215952 31042750 PMC6494051

[B52] Xie Z. Enkhjargal B. Reis C. Huang L. Wan W. Tang J. (2017). Netrin-1 preserves blood-brain barrier integrity through deleted in colorectal cancer/focal adhesion kinase/RhoA signaling pathway following subarachnoid hemorrhage in rats. J. Am. Heart Assoc. 6 (5), e005198. 10.1161/JAHA.116.005198 28526701 PMC5524080

[B53] Xiong Y. Mahmood A. Chopp M. (2013). Animal models of traumatic brain injury. Nat. Rev. Neurosci. 14 (2), 128–142. 10.1038/nrn3407 23329160 PMC3951995

[B54] Xu T. Zhang W. G. Sun J. Zhang Y. Lu J. F. Han H. B. (2015). Protective effects of thrombomodulin on microvascular permeability after subarachnoid hemorrhage in mouse model. Neuroscience 299, 18–27. 10.1016/j.neuroscience.2015.04.058 25936678

[B55] Yang C. Hawkins K. E. Doré S. Candelario-Jalil E. (2019). Neuroinflammatory mechanisms of blood-brain barrier damage in ischemic stroke. Am. J. Physiol. Cell Physiol. 316 (2), C135–C153. 10.1152/ajpcell.00136.2018 30379577 PMC6397344

[B56] Zhuge C. J. Zhan C. P. Wang K. W. Yan X. J. Yu G. F. (2022). Serum sulfonylurea receptor-1 levels after acute supratentorial intracerebral hemorrhage: Implication for prognosis. Neuropsychiatr. Dis. Treat. 18, 1117–1126. 10.2147/NDT.S368123 35685376 PMC9173726

